# Low-Cost MEMS Sensors and Vision System for Motion and Position Estimation of a Scooter

**DOI:** 10.3390/s130201510

**Published:** 2013-01-24

**Authors:** Alberto Guarnieri, Francesco Pirotti, Antonio Vettore

**Affiliations:** CIRGEO, Interdepartment Research Center for Geomatics, University of Padova, viale dell'Università 16, 35020 Legnaro, Padova, Italy; E-Mails: francesco.pirotti@unipd.it (F.P.);antonio.vettore@unipd.it (A.V.)

**Keywords:** Bayesian particle filter, Kalman filter, MEMS, Whippel model, motorcycle

## Abstract

The possibility to identify with significant accuracy the position of a vehicle in a mapping reference frame for driving directions and best-route analysis is a topic which is attracting a lot of interest from the research and development sector. To reach the objective of accurate vehicle positioning and integrate response events, it is necessary to estimate position, orientation and velocity of the system with high measurement rates. In this work we test a system which uses low-cost sensors, based on Micro Electro-Mechanical Systems (MEMS) technology, coupled with information derived from a video camera placed on a two-wheel motor vehicle (scooter). In comparison to a four-wheel vehicle; the dynamics of a two-wheel vehicle feature a higher level of complexity given that more degrees of freedom must be taken into account. For example a motorcycle can twist sideways; thus generating a roll angle. A slight pitch angle has to be considered as well; since wheel suspensions have a higher degree of motion compared to four-wheel motor vehicles. In this paper we present a method for the accurate reconstruction of the trajectory of a “Vespa” scooter; which can be used as alternative to the “classical” approach based on GPS/INS sensor integration. Position and orientation of the scooter are obtained by integrating MEMS-based orientation sensor data with digital images through a cascade of a Kalman filter and a Bayesian particle filter.

## 1. Introduction

The development of electronic systems for determining the position and orientation of moving objects in real-time has been a critical research topic for the last decade. Applications vary in many fields and range from rigid frames—aerial and land-based vehicles—as well as dynamic and complex frames like a human body [1,2]. The objective of measuring such parameters can also vary a lot; in remote sensing it is a crucial aspect for correct georeferencing of data acquired from optical sensors. Navigation and road safety purposes have also become common applications. Two main results of the technological progress in this field are represented by the Electronic Stability Program (ESP), an evolution of the Anti-Blocking System (ABS), and satellite positioning of vehicles. In the automotive sector, due to limited budgets and sizes, navigation sensors rely on the integration between a low cost GPS receiver and an Inertial Measurement Unit (IMU) based on Micro Electro-Mechanical System (MEMS) technology. Such integration is commonly realized through an extended Kalman filter [3–7], which provides optimal results for offsets, drifts and scale factors of employed sensors. However the application of this filter to motorcycle dynamics does not perform similarly. Unlike cars, motorcycles are able to rotate around their own longitudinal axis (roll), bending to the left and the right, therefore the yaw angular velocity is not measured by just one sensor, rather it is the result of the measurements of all three angular sensors, which contribute differently in time according to the current tilting of the motorcycle. Consequently, an error on the estimate of the roll angle at time t will affect the estimate of the pitch and yaw angles at next time t+1 as well. In this paper we consider the problem of detecting the position and orientation of a “Vespa”, a popular Italian scooter brand, using a low cost Positioning and Orientation System (POS) and images acquired by an on-board digital video camera. The estimate of the parameters (position in space and orientation angles) of the dynamic model of the scooter is achieved by integrating in a Bayesian particle filter the measurements acquired with a MEMS-based navigation sensor and a double frequency GPS receiver. In order to further improve the accuracy of orientation data, roll and pitch angles provided by the MEMS sensor are pre-filtered in a Kalman filter with those computed with the application of the cumulated Hough transform to the digital images captured by a video-camera.

In the next sections, after an overview of the system components, the method adopted for trajectory reconstruction is described in detail. Specifically, in Section 3 we present the “Whipple model” [8,9], which constitutes the mathematical basis of the dynamic model of the motorcycle, and in Section 4 we focus on the estimate of the roll angle from the images recorded by the video-camera using the cumulated Hough transform [10–12]. Then in Section 5 we discuss the use of the Bayesian particle filter to integrate MEMS sensor data with GPS measurements. Results achieved with the proposed method are reported in Section 6, while final conclusions are discussed in Section 7.

## 2. System Components

**Figure 1.**
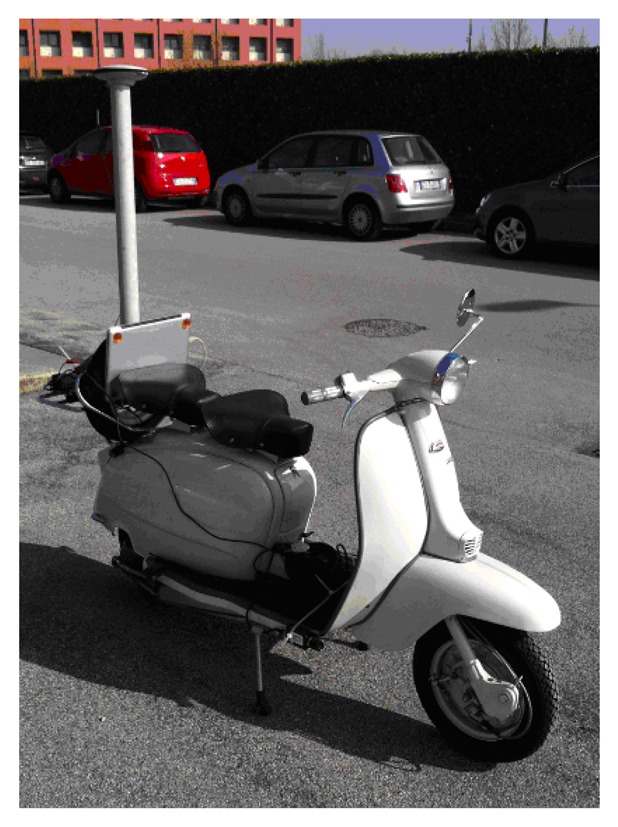
Side view of the Vespa scooter showing the data acquisition sensors. The digital video camera was placed on the right bottom side of the motorcycle.

The method for the motion estimation of a motorcycle proposed in this work has been tested on a “Vespa”, a common Italian scooter, which was equipped with a set of navigation sensors as shown in Figure 1. The system consists of an XSens MTi-G MEMS-based Inertial Measurement Unit (IMU) and a 1.3 Megapixel SONY Progressive Scan color CCD camera. The main technical specifications of the Xsens MTi-G are summarized in Table 1. Data acquisition and sensor synchronization were handled by a Notebook PC (Acer Travelmate) provided with 1,024 MB of RAM and a CPU processing speed of 1.66 GHz. A Novatel DL-4 double frequency GPS receiver was also fixed on the scooter and used to collect data for reference trajectory determination.

## 3. The Whipple Model

**Figure 2.**
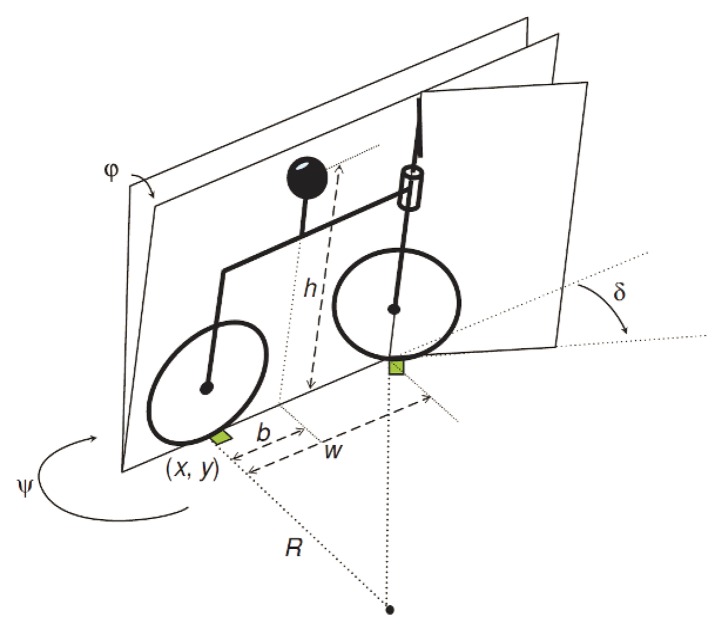
The inverted pendulum motorcycle model.

The “Whipple model” [8,9] consists in an inverse pendulum fixed in a frame moving along a line with the wheels which are considered to be discs with no width (Figure 2). The vehicle's entire mass *m* is assumed to be concentrated at its mass center, which is located at height *h* above the ground and distance *b* from the rear wheel, along the x axis. The parameter *ψ* represents the yaw angle, *φ* the roll angle, *δ* the steering angle and *w* is the distance between the two wheels. In this model the motorcycle motion is assumed to be constrained so that no lateral motion of the tires is allowed (non-holonomic constraint). The mathematical model does not take into account the possible oscillation of the scooter's wheel suspensions neither the movement of a driver. The motion equations are therefore described by:

(1) $$\begin{array}{l} \dot{x}=v\cos\psi \cos\theta \\ \dot{y}=v\cos\psi \cos\theta \\ \dot{z}=-v\sin\theta \end{array}$$

where x, y and z represent the real-time vehicle positions in the spatial frame, *v* is the forward velocity, and *θ* is the pitch angle (not shown in Figure 2).

From the geometry of the system the rate of change (*i.e.*, first derivative) of the yaw angle is defined as follows:

(2) $$\dot{\psi }=\frac{\tan\delta }{w\cos\varphi }v=\frac{v}{R}=\sigma v$$

where σ is the instantaneous curvature of the path followed by the motorcycle in the xy plane and R is the instantaneous curvature ray (σ = R^−1^).

According to the inverted pendulum dynamics; the roll angle satisfies the following equation:

(3) $$h\ddot{\varphi }=g\sin\varphi -\left[\left(1+h\sigma \sin\varphi \right)\sigma v^{2}+b\ddot{\psi }\right]\cos\varphi$$

where g is the acceleration due to gravity. The term *hσ sinφ* can be rewritten as a function of the steering angle *δ* and the roll angle *φ*:

(4) $$h\sigma \sin\varphi =\frac{h}{w}\tan\delta \tan\varphi$$

and given that angles *δ* and *φ* do not simultaneously assume high values, the term *hδ sin*φ can be neglected. Therefore, taking into account also Equation (2), Equation (3) becomes:

(5) $$h\ddot{\varphi }=g\sin\varphi -\left[\sigma v^{2}+b(\dot{v}\sigma +v\dot{\sigma })\right]\cos\varphi$$

Assuming that we can measure the roll angle *φ*(t), the pitch angle *θ*(t) and the velocity *v*(t), Equation (5) could be used to estimate the curvature *σ*. Indeed, by integrating Equation (5) we can compute the instantaneous curvature *σ*(t), provided that an initial condition *σ*(0) is given. Similarly, knowing the profile *σ*, if we integrate the non-holonomic kinematic model (1) from an initial position [x(0), y(0), z(0)] the path followed by the motorcycle can be fully reconstructed. In next sections we will discuss how we estimate the parameters *φ*, *θ* and *v*, whose knowledge is crucial for the application of the proposed method.

## 4. Roll and Pitch Angle Estimation

Roll and pitch angles can be estimated by using the frames recorded from the videocamera, which is rigidly fixed to the motorcycle, and detecting the position in the image of the horizon line estimating slope and distance of this line from the image origin. Using the perspective projection camera model, the horizon line projected onto the image plane can be described in terms of roll and pitch angles as follows (see [10,11] for details):

(6) cosθcosφV−sinφU=sinθcosφ

where (U,V) denote the image plane coordinates of a point P with coordinates [x,y,z] in the camera frame Σ_c_. Therefore, the pitch and roll angles *θ* and *φ* can be determined knowing the position of the horizon line in the image. Despite the horizon cannot be easily determined due to occlusions frequently occurring in the scene, roll and pitch rates can be robustly estimated by comparing two consecutive images. Indeed, given the horizon line in the frame at time t_i_, I(t_i_), in the next frame at time t_i_+1, I(t_i_+1), the horizon is described by the following relationship:

(7) cos(θ+Δθ)cos(φ+Δφ)V−sin(φ+Δφ)U=sin(θ+Δθ)cos(φ+Δφ)

Linearizing Equation (7) about *θ*(t) and *φ*(t), neglecting terms of order higher than one in Δ and assuming small pitch angles (*θ* ≅ *t*), we obtain:

(8) sinφΔφV+cosφΔφU=−Δθcosφ

Equation (8) shows that in two successive frames, the horizon rotates by *Δφ* and translates by −*Δθ* cos*φ*. These two quantities (*Δφ*, *Δθ*) can be measured by computing the Hough transform on a region of interest centered around a neighborhood of the current estimation of the horizon line.

The Hough transform [12] is a feature extraction technique used in image analysis, computer vision, and digital image processing, whose purpose is to find imperfect instances of objects within a certain class of shapes by a voting procedure. This voting procedure is carried out in a parameter space, from which object candidates are obtained as local maxima in a so-called accumulator space that is explicitly constructed by the algorithm for computing the Hough transform. In this case this transform is used to determine the horizon line in the images acquired by the scooter's on-board videocamera. To this aim polar coordinates (*ρ*,*α*) are used as space parameters and are related to the image coordinates (U,V) as follows:

(9) ρ=Ucosα+Vsinα

**Figure 3.**
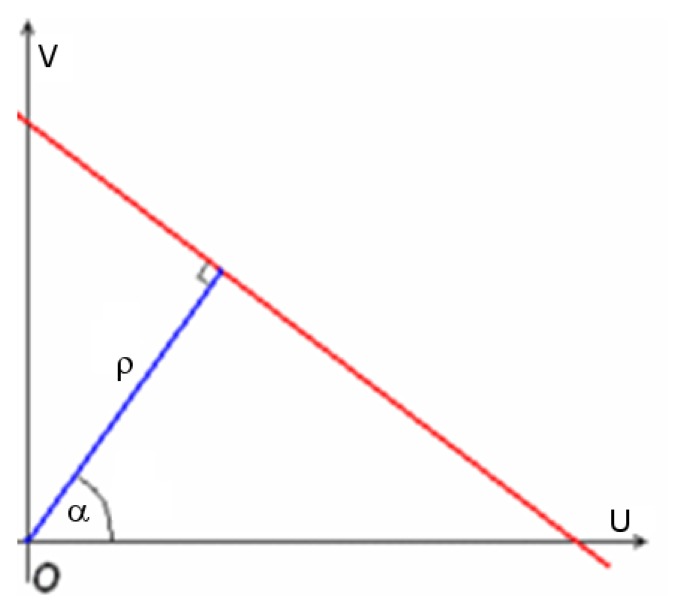
The parameter space (*ρ*,*α*) of the Hough transform adopted for line detection.

An example of such image space parametrization is shown in Figure 3.

**Figure 4.**
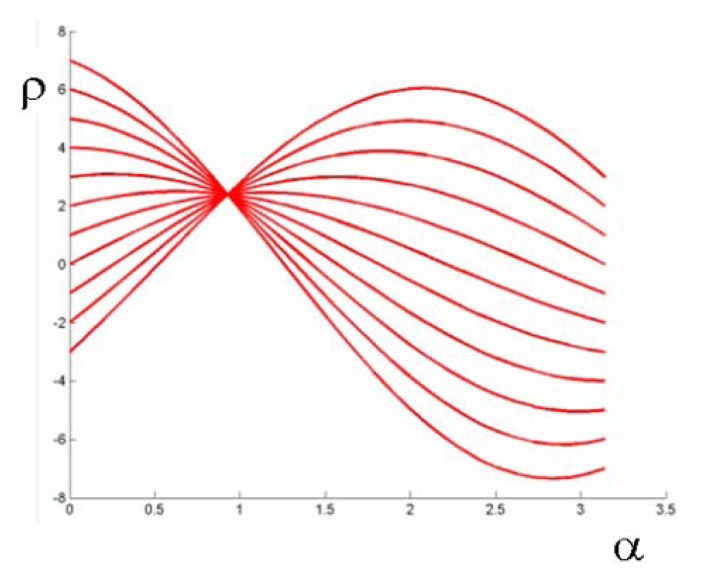
Image points mapped into the parameter space.

Given this parameterization, points in parameter space *ρ*, *α*) correspond to lines in the image space, while points in the image space correspond to sinusoids in parameter space, and *viceversa* (Figure 4). The Hough transform allows therefore to determine a line (e.g., the horizon) in the image as intersection, in parameter space, of sinusoids corresponding to a set of co-linear image points. Such points can be obtained by applying an edge detection algorithm.

The steps needed to compute the rates (*Δφ*, *Δθ*) can be summarized as follows:

1. Apply an edge detection to a predefined region of interest of the image;
2. Perform a discretization the parameter space (*ρ*, *α*) by subdividing it in a set of cells (bins);
3. Considering that each edge candidate is an infinite line segment of polar coordinates (*ρ*, *α*), the number of edges falling in each bin is counted;
4. Through this accumulation an histogram of an image in coordinates (*ρ*, *α*) is generated, whose intensity values are proportional to the number of edges falling in each bin. This histogram represents the Hough transform H(*ρ*, *α*) of the image.

From each histogram the corresponding cumulated Hough transform is derived. This transform is a modification of the Hough transform and is defined as follows:

(10) $${\bar{\text{H}}}_{\text{E}}\;(\alpha )=\sum_{\rho }{\text{H}}_{\text{E}}\;(\rho ,\alpha )$$

Equation (10) holds for the roll angle (*α* = *φ*), while for the pitch angle (*α* = *θ*) it becomes:

(11) $${\bar{\text{H}}}_{\text{E}}\;(\rho )=\sum_{\alpha }{\text{H}}_{\text{E}}\;(\rho ,\alpha )$$

**Figure 5.**
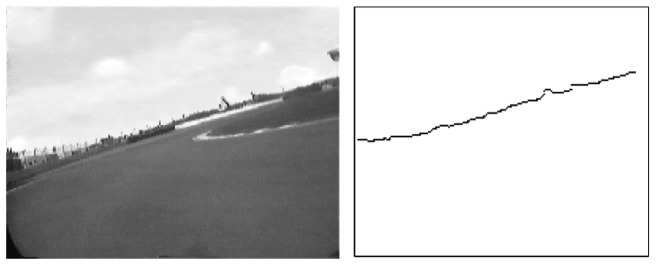
(**Left**): Image acquired from the on-board camera. (**Right**): edge detection of the horizon line.

**Figure 6.**
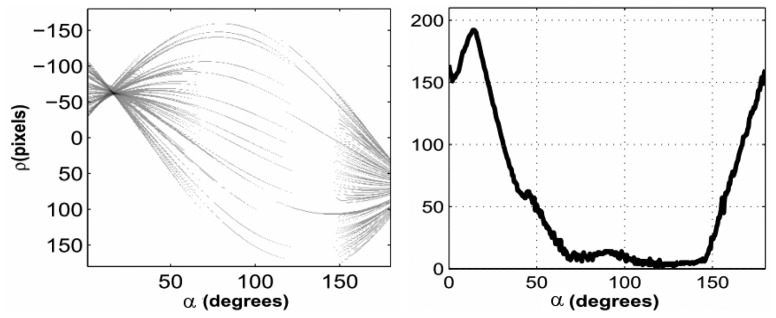
(**Left**): Hough transform obtained from the set of edges in Figure 5. (**Right**): corresponding cumulated Hough transform.

**Figure 7.**
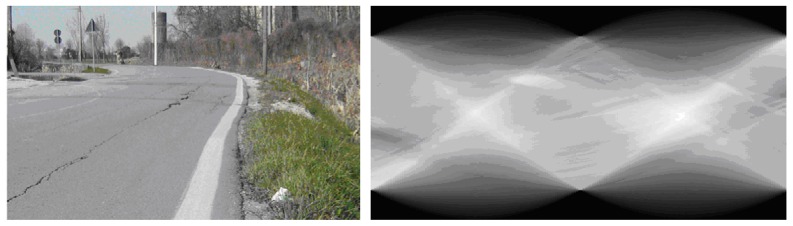
(**Left**): Image acquired during a drive test. (**Right**): Corresponding Hough transform.

An example of the cumulated Hough transform is shown in Figures 5–7.

It can be proved that if the same edges are visible at time t and t+ Δt, then for the roll angle (and similarly for the pitch angle) it holds that:

(12) $$\begin{array}{cc} {\bar{\text{H}}}_{\text{E}(t+\Delta t)}(\varphi )={\bar{\text{H}}}_{\text{E}(t)}(\varphi +\Delta \varphi (\text{t})) & \forall \varphi \in [0,\pi ) \end{array}$$

In presence of noise and considering that not all edges visible at time *t* remain visible at time *t*+Δ*t*, a good estimation of *Δφ*(*Δt*) can be obtained minimizing the Euclidean distance between each of the cumulated transforms at time *t* and *t*+*Δt*:

(13) $$\Delta \varphi (\Delta t)=\underset{\Delta \alpha }{\arg\min}\|\int H_{t+\Delta t}\;(\rho ,\alpha -\Delta \alpha )d\rho -\int H_{t}\;(\rho ,\alpha )d\rho \|$$

Similarly, the estimate of the increment of the roll angle *θ* is computed as follows:

(14) $$\Delta \theta (\Delta t)=\frac{1}{\cos\varphi }\underset{\Delta \rho }{\arg\min}\|\int H_{t+\Delta t}\;(\rho -\Delta \rho ,\alpha )d\alpha -\int H_{t}\;(\rho ,\alpha )d\alpha \|$$

After these steps, the estimates of the roll and pitch angles are computed by time integration of the rates *Δφ* and *Δθ*.

## 5. The Bayesian Particle Filter

The key point of all navigation and tracking applications is the motion model to which bayesian recursive filters (as the particle filter [13]) can be applied. Models which are linear in the state dynamics and non-linear in the measurements can be described as follows:

(15) $$\begin{array}{l} x_{t+1}={\text{Ax}}_{t}+B_{u}\;u_{t}+B_{f}\;f_{t}\\ y_{t}=h(x_{t})+e_{t} \end{array}$$

where x_t_ is the state vector at time *t*, u_t_ the input, f_t_ the error model, y_t_ the measurements and e_t_ the measurement error. In this model, indipendent distributions are assumed for f_t_, e_t_ and the initial state x_0_, with known probability densities p_et_ , p_ft_ and p_x0_, respectively, but not necessarily Gaussian.

We denote the set of available observations at time *t* as:

(16) $$Y_{t}=\left\{y_{0},\ldots ,y_{t}\right\}$$

**Figure 8.**
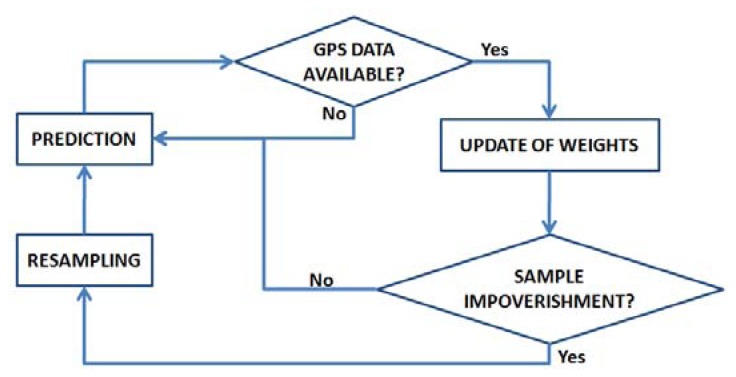
Block diagram of the Bayesian particle filter.

The Bayesian solution to equations (15) deals with the computing of the a prior distribution p(x_t+1_|Y_t_), given past distribution p(x_t_|Y_t_). In case the noise can be modeled by indipendent, white and gaussian with zero mean probability density functions, and h(x_t_) is a linear function, then the optimal solution is provided by the Kalman filter. Should be this condition not satisfied, an approximation of the a prior distribution p(x_t+1_|Y_t_) can be still provided using a Bayesian particle filter [13]. This filter is an iterative process by which a collection of particles, each one representing a possible target state, approximates the a prior probability distribution, which describes the possible states of the target. Each particle is assigned a weight *w*_t_^i^, whose value will increase as closer to true value the related sample will be. When a new observation arrives, the particles are time updated to reflect the time of the observation. Then, a likelihood function is used to updated the weights of the particles based on the new information contained in the observation. Finally, resampling is performed to replace low weight particles with randomly perturbed copies of high weight particles. A block diagram of the particle filter is presented in Figure 8.

Since the computational cost of a particle filter is quite high, only an adequate minimum number of variables has been included in the dynamic model of the scooter. It was therefore chosen to neglect any movement along the z axis (e.g., “bouncing” of suspensions), and to account for position variables x and y, speed *v*, the three angles needed for modelling the orientation (*φ*, *θ*, *ψ*) and the filtered version of the curvature *σ*. In order to further improve the accuracy of orientation data, roll and pitch angles provided by the MEMS sensor have been combined and pre-filtered in a Kalman filter with those computed using the cumulated Hough transform applied to the digital images captured by a video-camera. Assuming that the system is now represented as a collection of N particles, the dynamics of the generic particle s^i^ (*i.e.*, a possible system state) is described by the following model:

(17) $$\{\begin{array}{ll} x_{t+1}^{i} & =x_{t}^{i}+v_{t}^{i}\cos(\psi_{t}^{i})\cos(\theta_{t}^{i})\Delta T+N(0,\Delta x_{t}^{i2})\\ y_{t+1}^{i} & =y_{t}^{i}+v_{t}^{i}\sin(\psi_{t}^{i})\cos(\theta_{t}^{i})\Delta T+N(0,\Delta y_{t}^{i2})\\ v_{t+1}^{i} & =v_{t}^{i}+(a_{t}-g\cos(\theta_{t}^{i})\Delta T+N(0,\Delta v_{t}^{i2})\\ \varphi_{t+1}^{i} & =(1-\gamma_{r_{t}}^{i})(\varphi_{t}^{i}+\varphi_{t}^{i}\Delta T)+\gamma_{r_{t}}^{i}\arctan\left(\frac{\sigma_{f_{t}}^{i}v_{t}^{i2}}{g}\right)\\ \theta_{t+1}^{i} & =\theta_{t}^{i}+{\dot{\theta }}_{t}^{i}\Delta T\\ \psi_{t+1}^{i} & =\psi_{t}^{i}+{\dot{\psi }}_{t}^{i}\Delta T+N(0,\Delta \psi_{t}^{i2})\\ \sigma_{f_{t+1}}^{i} & =(1-\gamma_{s})\sigma_{f_{t}}^{i}+\gamma_{s}\frac{\psi_{t}^{i}}{v_{t}^{i}}\\ w_{t+1}^{i} & =\frac{w_{t}^{i}P_{t}(p_{t}^{i})}{\sum_{j=1}^{N}w_{t}^{i}P_{t}(p_{t}^{j})} \end{array}$$

where:

- ΔT is the sampling interval;
- N(0, Δx^i2^_t_) represents the measurement noise of the X coordinate, modeled as a Gaussian function with a zero mean and standard deviation Δx^i^_t_. Similar assumption holds for measurement noises N(0,Δy^i2^_t_), N(0,Δv^i2^_t_) and N(0,Δψ^i2^_t_);
- $\sigma_{f_{t+1}}^{i}$ is the weighted combination of the curvature estimated at previous time $t\left(\sigma_{f_{t}}^{i}\right)$ and the current input $\frac{\psi_{t}^{i}}{v_{t}^{i}}$, being *γ*_s_ the weighting term (*γ*_s_ = 1/10);
- $w_{t}^{i}$ is weight of the i-th particle;
- $P_{t}(s_{t}^{i})$ is the *importance* function, *i.e.*, the likelihood function through which the weights are updated according to the following relationship:
  (18) $$w_{t+1}^{i}=w_{t}^{i}P(y^{t}|x_{t}^{i})$$
- $\gamma_{r_{t}}^{i}$ is a coefficient which dynamically changes in order to give more weight to minimal curvatures and roll angular velocities as denoted by:
  (19) $$\gamma_{r_{t}}^{i}=\{\begin{array}{cc} \gamma_{m}\left(\sigma_{l}-\left|\sigma_{f_{t}}^{i}\right|\right)\left({\dot{\varphi }}_{l}-\left|{\dot{\varphi }}_{t}^{i}\right|\right) & if\left|\sigma_{f_{t}}^{i}\right|<\sigma_{l}\;\text{and}\;\left|{\dot{\varphi }}_{t}^{i}\right|<{\dot{\varphi }}_{l}\\ 0 & \text{otherwise} \end{array}$$
  where *σ_l_* and *φ̇_l_* are the thresholds for the maximum curvature and roll angular velocity respectively. We set *γ_m_* = 1/500, *σ_l_* = 1/100 m^−1^ and *φ̇_l_* = 30 °/s.

In the model Equation (17) we used different formulas for the derivatives of the orientation angles ${\dot{\varphi }}_{t}^{i}$, ${\dot{\theta }}_{t}^{i}$ and ${\dot{\psi }}_{t}^{i}$. This is due to the fact that the angular velocities (ω_x_, ω_y_, ω_z_) measured by the MEMS sensor are related to the *body* frame (*i.e.*, the coordinate system fixed with the scooter) while orientation angles (*φ*, *θ*, *ψ*) are determined in a *world* reference frame (e.g., the GPS coordinate system, WGS-84). A frame transformation from the *body* to the *world* frame is therefore needed, which leads to different equations for the orientation angles.

The components of the state vector at time *t* are then computed as weighted average of the variables estimated by the filter, using the weights *w*^i^ of all particles *s*^i^:

(20) $${\left(\begin{array}{lllllll} x_{t} & y_{t} & v_{t} & \varphi_{t} & \theta_{t} & \psi_{t} & \sigma_{t} \end{array}\right)}^{T}=\sum_{i=1}^{N}w_{t}^{i}{\left(\begin{array}{lllllll} x_{t}^{i} & y_{t}^{i} & v_{t}^{i} & \varphi_{t}^{i} & \theta_{t}^{i} & \psi_{t}^{i} & \sigma_{f_{t}}^{i} \end{array}\right)}^{T}$$

In order to limit the computational effort of the filter, the update of the particle weights w_i_ is not performed at every step of the algorithm, but rather when the GPS data are available from the receiver.

## 6. Results and Discussion

**Figure 9.**
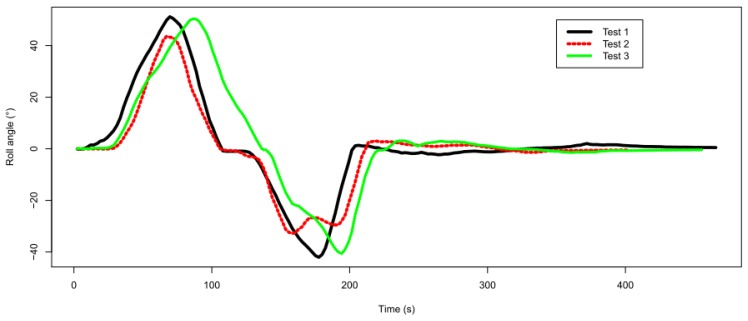
Roll angle profiles resulting from the performed tests.

Three drive tests were carried out on the same track in order to evaluate the measurement repeatability, whose results for the roll angle are shown in Figure 9. A slight difference can be observed for test No. 3 where the speed was slower than for the other two tests.

**Figure 10.**
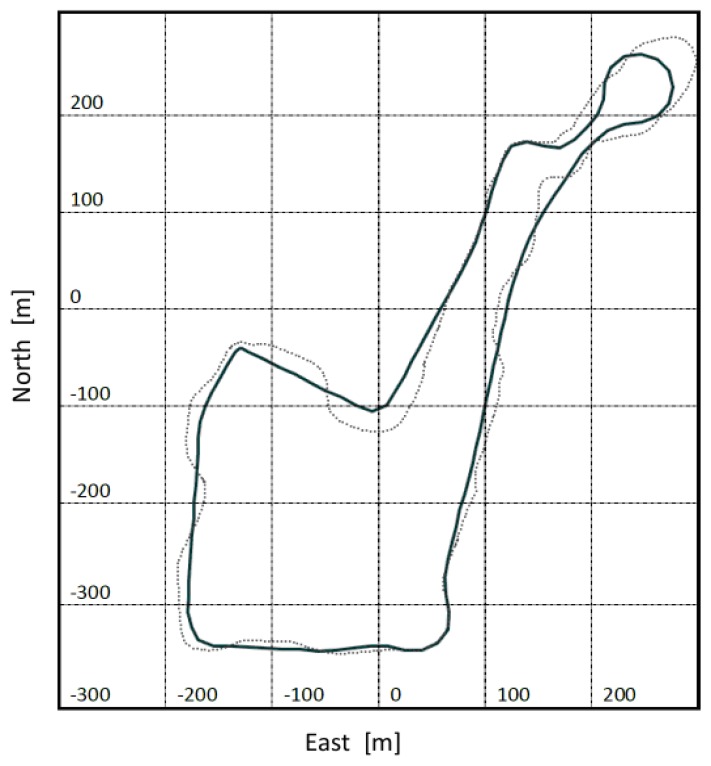
The estimated trajectory (dotted line) overimposed onto the GPS reference track (solid line).

An example of the track reconstructed from the data received during one of the three tests is shown in Figure 10. Here the trajectory (dotted line) estimated with the Bayesian particle filter is compared with the reference trajectory derived from differentially corrected GPS measurements (solid line). Beyond vibrations, offsets and scale factors, further interesting sources of error to be tested are wrong initial conditions and noises of the roll and pitch angles. During the test the roll angle was brought to more than 20° to evaluate the performance of the filter; no GPS update was used by the Bayesian particle filter to estimate the trajectory covered by the scooter. The algorithm was able to converge, albeit slowly, towards the real angle. Some statistics highlighting the residual distances between the reference trajectory shown in Figure 10 and that estimated with the particle filter are summarized in Table 2.

Developments of the proposed method will deal with the encoding of the Bayesian filter inside an integrated system which can be used to equip the scooter. This can lead in the future to provide even motorcycles with traction control systems. Further developments will be the inclusion in the dynamic model of the suspensions' motion along the Z axis, and also the study of the influence of the steering angle (δ) on the estimation of the roll angle. These two parameters are indeed related by the following relationship, which can be easily derived from Equation (2):

(21) $$\varphi =\text{arc}\cos\left(\frac{\tan\delta }{W\sigma }\right)$$

## 7. Conclusions

In this paper we have presented an alternative method for the reconstruction of the trajectory of a motorcycle (“Vespa” scooter) with respect to the “classical” approach based on GPS/INS sensor integration. In our implementation position and orientation of the scooter are obtained by integrating MEMS-based orientation sensor data with digital images through a cascade of a Kalman filter and a Bayesian particle filter. As shown, the proposed method provides quite acceptable results though its application is affected by environment conditions. Indeed the roll angle estimation based on the Hough transform requires a minimal amount of linear elements in the scene, whose absence can degrade the results achievable for the roll angle. For example a complex skyline and low contrast between the road segment and neighboring object can be problematic, even if not common.

## Figures and Tables

**Table 1.** Main technical specifications of Xsens MTi-G.

Static accuracy (roll/pitch)	<0.5 deg

Static accuracy (heading)	<1 deg

Dynamic accuracy	1 deg RMS

Angular resolution	0.05 deg

Dynamic range:	
- Pitch	±90deg
- Roll/Heading	±180 deg

Accuracy position (SPS)	2.5 m CEP

Maximum update rate:	
- Onboard processing	120 Hz
- External processing	512 Hz

Dimensions	58 × 58 × 33 mm (W × L × H)

Weight	68 g

Ambient temperature (operating range)	−20 … +55 °C

**Table 2.** Statistics of the residual displacements btw. the GPS reference trajectory and that estimated by the particle filter shown in Figure 10.

Minimum	0.042 m
Maximum	10.116
Mean	1.033
Absolute mean	3.2 m
Standard deviation (operating range)	2.533 m
